# Cerulenin Blockade of Fatty Acid Synthase Reverses Hepatic Steatosis in ob/ob Mice

**DOI:** 10.1371/journal.pone.0075980

**Published:** 2013-09-27

**Authors:** Gang Cheng, Arun P. Palanisamy, Zachary P. Evans, Alton G. Sutter, Lan Jin, Inderjit Singh, Harold May, Michael G. Schmidt, Kenneth D. Chavin

**Affiliations:** 1 Divisions of Transplant Surgery, Department of Surgery, Medical University of South Carolina, Charleston, South Carolina, United States of America; 2 Department of Pediatrics, Medical University of South Carolina, Charleston, South Carolina, United States of America; 3 Department of Microbiology and Immunology, Medical University of South Carolina, Charleston, South Carolina, United States of America; UAE University, Faculty of Medicine & Health Sciences, United Arab Emirates

## Abstract

Fatty liver or hepatic steatosis is a common health problem associated with abnormal liver function and increased susceptibility to ischemia/reperfusion injury. The objective of this study was to investigate the effect of the fatty acid synthase inhibitor cerulenin on hepatic function in steatotic *ob/ob* mice. Different dosages of cerulenin were administered intraperitoneally to *ob/ob* mice for 2 to 7 days. Body weight, serum AST/ALT, hepatic energy state, and gene expression patterns in *ob/ob* mice were examined. We found that cerulenin treatment markedly improved hepatic function in *ob/ob* mice. Serum AST/ALT levels were significantly decreased and hepatic ATP levels increased in treated obese mice compared to obese controls, accompanied by fat depletion in the hepatocyte. Expression of peroxisome proliferator-activated receptors α and γ and uncoupling protein 2 were suppressed with cerulenin treatment and paralleled changes in AST/ALT levels. Hepatic glutathione content were increased in some cases and apoptotic activity in the steatotic livers was minimally changed with cerulenin treatment. In conclusion, these results demonstrate that fatty acid synthase blockade constitutes a novel therapeutic strategy for altering hepatic steatosis at non-stressed states in obese livers.

## Introduction

Hepatic steatosis or fatty liver is an increasingly common health problem due to accumulation of fat within hepatocytes. In Western countries, up to one third of the general population is affected by hepatic steatosis [1], [2], [3], and is now the most common cause of chronic liver disease in children and adolescents [4]. Hepatic steatosis has been associated with increased sensitivity of the liver to other injuries, such as insulin resistance [2] and ischemia/reperfusion (I/R) during transplantation [5]. A substantial number of patients with hepatic steatosis may finally develop cirrhosis [3], [6]. This raises an even greater public health concern as it is recognized that cirrhosis has a 10-year liver-related mortality of 25% [1], [3].

Lipid accumulation inside of hepatocytes is associated with a number of intracellular disorders including mitochondrial abnormalities [7], impaired ability to synthesize ATP [8], [9], and increased generation of reactive oxygen species (ROS) [10], [11]. Uncoupling protein 2 (UCP2), which uncouples mitochondrial oxidative phosphorylation and thus depletes intracellular ATP, has been associated with hepatic steatosis [9]. We have previously demonstrated that UCP2 expression in the livers of obese *ob/ob* mice is markedly increased, which may contribute to a high mortality and slower recovery from I/R injury [12]. Accumulating evidence suggests that fatty acids induce UCP2 expression in hepatocytes [12], [13], [14], and this activation could be mediated by peroxisome proliferator-activated receptors (PPARs) [13], [15]. In addition, increased production of ROS has been observed in steatotic livers [7], [16], which may be responsible for the progression from steatosis to cirrhosis [17], [18].

Excess fat plays a central role in the pathogenesis of hepatic steatosis, and that the effects of excess fat are mediated by PPARs and UCP2 overexpression [13]. The natural product cerulenin ([2R,3S]-2,3-epoxy-4-oxo-7,10-trans,trans-dodecadienamide) is a mycotoxin originally developed as an antifungal antibiotic but has potent inhibitory effect on fatty acid synthase (FAS) [19], [20]. Cerulenin binds covalently to a cysteine residue at the active site of the condensing enzyme and irreversibly inhibits β-ketoacyl-ACP synthase activity of FAS [21], [22]. Previously, cerulenin has been shown to cause sustained weight loss and decreased fat pad size [23], [24], [25] and increase viability following I/R in *ob/ob* mice [26]. The objectives of this study were to investigate whether and how cerulenin improves steatotic liver function in *ob/ob* mice, in the basal non-stressed state.

## Materials and Methods

### Ethics Statement

The use of animals is necessary in this study because of the nature of information sought. All rodents used for surgeries were initially anesthetized using isoflurane in desiccators then followed by isoflurane as needed. Animals were observed post-operatively for signs of distress as in respiratory distress, blood pressure, and discernable pain. Buprenorphine was given as an analgesic drug to reduce pain and discomfort. Animals are removed from the study and euthanized by exsanguination (under anesthesia) or CO2 when clearly suffering negates the need to continue humanely in accordance with the Medical University of South Carolina’s Institutional Animal Care and Use Committee (IACUC) policy. This study was reviewed and approved by the Medical University of South Carolina’s IACUC (AR# 3003: Effects of Steatosis on Ischemia/Reperfusion and Liver Regeneration).

### Animals

*ob/ob* mice (Jackson Laboratory, ME) are obese with hepatic steatosis as a result of homozygous leptin gene deficiency. Mice were housed 3 to 4 per cage in a pathogen-free temperature-controlled room (22–25°C) with a 12-hour light-dark cycle and provided with water and food available *ad libitum*. All animals received humane care and all animal experiments have been reviewed and approved by the university Institutional Animal Care and Use Committee (IACUC).

### Cerulenin Treatment

Cerulenin (Sigma) was given to 6–8 week old male *ob/ob* mice in RPMI medium containing 20% DMSO intraperitoneally (*i.p*.) as previously described [24], [27]. Controls were injected similarly with vehicle alone. The experimental groups (4 mice each) were as follows: A: 60 mg/kg/day cerulenin, injected daily for 7 days; B: 60 mg/kg every other day for 7 days; C: 30 mg/kg/day for 7 days; D: 30 mg/kg every other day for 7 days; E: vehicle, daily for 7 days; F: 60 mg/kg/day cerulenin for 2 days; G: 30 mg/kg/day cerulenin for 2 days; H: vehicle, daily for 2 days; I: control. All animals were sacrificed on the same day under anesthesia. Blood was collected by portal vein puncture. Liver samples were snap-frozen in liquid N_2_ and stored at –80°C until analysis, or paraformaldehyde-fixed for histological analysis.

### Serum Aminotransferases

Collected blood was allowed to clot at room temperature for 15 minutes, centrifuged at 3500 g for 5 minutes to collect serum, and evaluated for AST/ALT by a Coulter counter from the university Clinical Laboratory Services.

### ATP Assay

Cellular ATP content was analyzed in triplicate as described previously [28]. For normalization between samples, total cellular protein from each sample of tissue homogenate was determined by BCA assay.

### Northern Blot Analysis

Murine UCP2 cDNA was a gift from Dr. Paul Dowell (Johns Hopkins University). Mouse PPARα and γ cDNAs were made by RT-PCR according standard protocols, and the primer sequences are available upon request. Total cellular RNA preparations and Northern blot were performed as described previously [28]. For normalization, the blots were stripped and re-hybridized with 18 S RNA cDNA probe.

### Immunohistochemistry

Rabbit anti-PPARγ antibody (Alexis Biochemicals, Germany, 1∶1000 dilution) was used to detect PPARγ expression in the liver in paraformaldehyde-fixed, paraffin-embedded liver sections according to standard techniques [29].

### Histochemical Analysis

H&E staining was performed in paraffin-embedded liver sections, while Oil-Red-O staining (ORO) staining was used on frozen sections to avoid fat solvents used in processing paraffin sections, according to standard protocols.

### Gas Chromatography-flame Ionization Detection (GC-FID) Analysis of Lipid Content

Fatty acid (FA) from liver samples were extracted as described before [30]. Liver homogenates were separated by organic separation filter. BF3-10% methanol was added to catalyze the methanolysis of the fats to FA methyl esters and an internal standard was added before each run. Samples (1 µl) were injected by an Agilent auto sampler into an RTX-624 capillary column of a Hewlett-Packard 5890 GC (Agilent).

Column temperature was held at 40°C for 2 min, followed by a temperature ramp of 20°C/min to 315°C where the column was held for a minimum of 5 minutes. A FA methyl esters standard mix was used to standardize the instrument and analyze the samples.

### GSH Assay

Micro titer plate assay for the measurement of glutathione and glutathione disulfide was done as described previously [31]. All reagents were from Sigma.

### In Situ Detection of Apoptosis

TUNEL assay was done in paraformaldehyde-fixed, paraffin-embedded liver sections according to standard techniques [32].

### Statistical Analysis

Statistical significance was determined by chi-square test for viability assay, and by a two-tailed Student’s *t* test for all other analyses. Data are presented as the mean±SD and a *p* value of <0.05 was considered statistically significant.

## Results

### Cerulenin Decreases Weight Gain in *ob/ob* Mice

**Figure 1:**
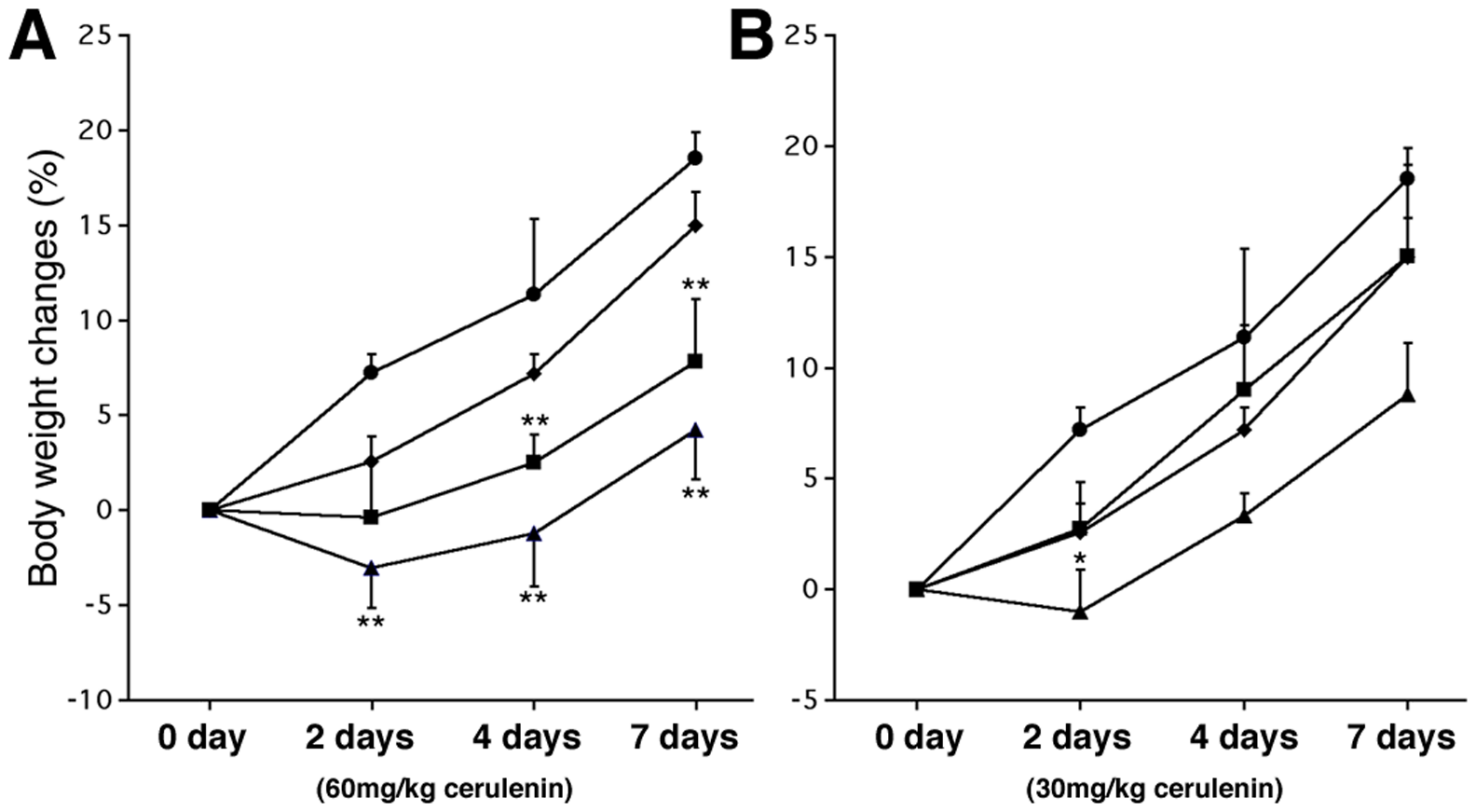
Body weight changes with cerulenin treatment. The control (circle) and the vehicle alone (diamond) are shown in both panels. Panel **A** shows the body weight changes of the groups with 60 mg/kg cerulenin, given either daily (triangle) or every other day (square), panel **B** shows the groups with 30 mg/kg cerulenin, given either daily (triangle) or every other day (square). Statistical analysis in each experimental group was compared with the vehicle treated group. *: *P<0.05*; **: *P<0.01*.

Cerulenin treatment of *ob/ob* mice had obvious effects on body weight. With 2 days of treatment, body weight in treated mice was decreased compared to a 5.7% weight gain in the controls. With prolonged (7 days) treatment, no body weight loss was observed, but body weight gain was slowed. In all groups, 60 mg/kg of cerulenin was more effective than 30 mg/kg in inhibiting weight gain **(** **Fig. 1** **).**

### Cerulenin Improves Liver Function in *ob/ob* Mice

**Figure 2:**
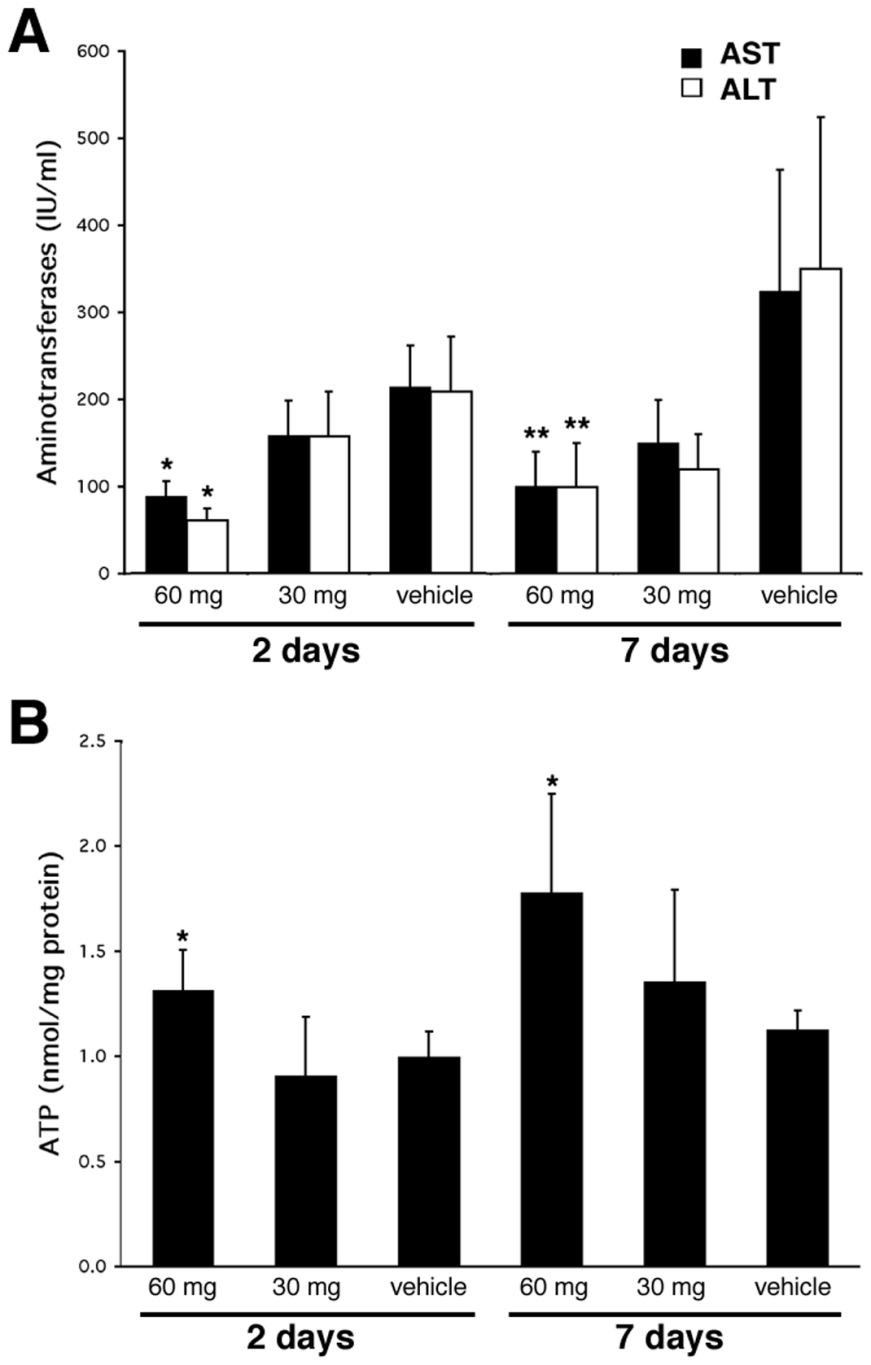
Serum AST/ALT and ATP contents. Panel **A**, serum AST/ALT changes with 2 days or 7 days of daily cerulenin treatment. Panel **B**, ATP content with 2 days or 7 days of daily cerulenin treatment. *: *P<0.05*; **: *P<0.01*.

ob/ob mice usually have baseline hepatitis as evidenced by increased AST/ALT levels. In our experiments, we found that there was no difference in serum aminotransferase (AST/ALT) levels between baseline control and vehicle-treated mice (data not shown). However, 60 mg/kg cerulenin, given either daily or every other day for 7 days, significantly reduced both AST and ALT in treated mice compared with 7-day vehicle-treated controls, with serum AST/ALT levels decreased 69.1% and 71.8% respectively after daily treatment **(** **Fig. 2A** **)**, and decreased 69.8% and 78.5% respectively after every other day treatment. It is interesting to note that cerulenin at 60 mg/kg/day given for 2 days was as effective as 7 days of treatment in improving liver function, with serum AST/ALT levels decreased by 53.8% and 66.1% respectively in treated mice compared with 2-day vehicle-treated controls **(** **Fig. 2A** **)**. In contrast, 30 mg/kg cerulenin at all dosage regimes decreased AST and ALT but did not achieve any statistical significance **(** **Fig. 2A** **).**

### Cerulenin Increases ATP Level in the Livers of *ob/ob* Mice

We have previously demonstrated that ATP levels in steatotic mice are lower than lean mice [9], [33]. Given an improvement in hepatic function in the *ob/ob* mice with FAS blockade, we next sought to determine if this would also affect ATP content. We found that if given daily or every other day, ATP content were increased 58.1% and 61.5% respectively by 7-day treatment of 60 mg/kg cerulenin (**Fig. 2B**). Significant ATP elevation was also observed with only 2 days of treatment with 60 mg/kg cerulenin (**Fig. 2B**). In contrast, 30 mg/kg cerulenin, given either 2 or 7 days, did not show any significant effect on cellular ATP content.

### Cerulenin Decreases Fat Content and Alters the Fat Type

**Figure 3:**
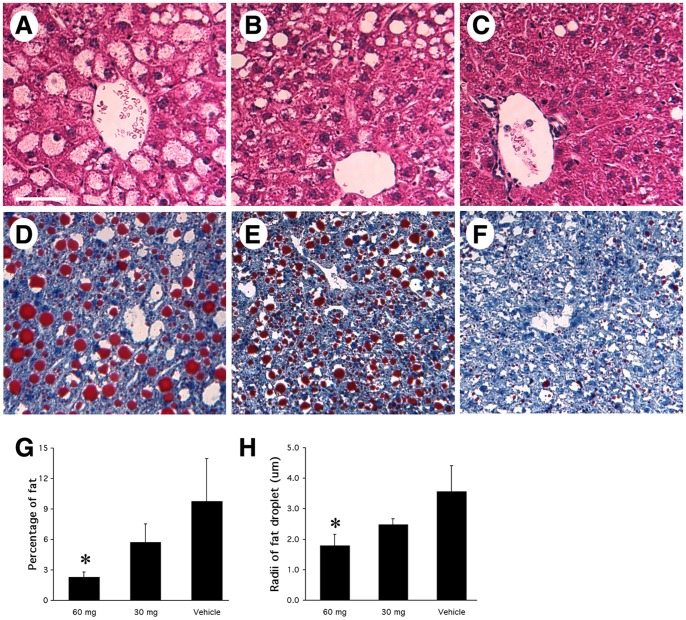
Histochemical analysis of *ob/ob* mouse livers with cerulenin treatment. Panels **A–C**: H&E staining. **A.** vehicle-treated *ob/ob* mouse liver, shows most hepatocytes had a big fat droplet (pale staining, representing the empty space left behind when the fat is removed during tissue processing), which occupied most space in the hepatocytes, while the nuclei was localized to the periphery. **B**. cerulenin-treated (60 mg/kg/day for 2 days) *ob/ob* mouse liver, shows significantly decreased number and size of fat droplets. In most hepatocytes, microsteatosis dominated. **C.** cerulenin-treated (60 mg/kg/day for 7 days) *ob/ob* mouse liver. Except for a few fat droplets, the overall appearance of the liver was almost to normal, especially in the areas close to the central vein. Panels **D–F**: Oil-Red-O staining of *ob/ob* mouse liver with 7 days of treatment; Red signals represent fat droplets. The treatment was: **D**. vehicle only; **E.** cerulenin-treated (60 mg/kg/day) for 2 days; **F.** cerulenin-treated (60 mg/kg/day) for 7 days. **G**: percentage of fat content in liver cross sections with 7-day treatment of either 60 or 30 mg/kg/day cerulenin. **H**: size of fat droplets in liver cross sections with 7-day treatment of either 60 or 30 mg/kg/day cerulenin. Scale bar: 60 µm. *: *P<0.05.*

The gross appearance of cerulenin treated steatotic livers improved. While fatty livers in the control group were yellowish, pale, and larger in size, cerulenin treated steatotic livers looked dark-brown and smaller, a phenotype more consistent with non-fatty (normal) livers. H&E staining of these specimens revealed that fat content in cerulenin-treated livers was much less than the controls (a phenomenon not observed in vehicle-treated controls), with the most obvious morphological improvement in the centrilobular zone **(** **Fig. 3A–C** **)**. To analyze changes in fat content in these samples, ORO was performed on freshly frozen liver sections. The histological profile of *ob/ob* livers that received cerulenin treatment demonstrated a dramatic reduction in lipid content, which was dose and time dependent. Seven-day treatment of 60 mg/kg cerulenin daily or every other day demonstrated the greatest decrease of fat staining and in some areas of the liver, ORO staining was absent **(** **Fig. 3D–F** **).** Two-day treatment of 60 mg/kg cerulenin resulted in a modest reduction in fat content. In contrast, cerulenin given 30 mg/kg was less effective than 60 mg/kg doses, but its effect on fat content was still obvious except for 2-day treatment (data not shown).

Generally, the steatosis observed in the control animals was predominantly macrovesicular, and the hepatocyte nuclei were displaced into the periphery of the cell by a large fat droplet. Upon treatment with cerulenin, the size of the fat droplets decreased, suggesting a conversion from macrosteatosis to microsteatosis **(** **Fig. 3D–F** **)**. The computer program NIH Image was employed for analysis of fat content in 7-day treated livers. The percentage of fat observed in the liver cross-sections decreased from an average of 9.77% ±4.2% in the vehicle control to 2.29% ±0.5% in the animals treated with 60 mg/kg/day of cerulenin (p<0.05) and 5.75% ±1.8% with 30 mg/kg/day cerulenin **(** **Fig. 3G** **)**. Similarly, we analyzed the radii of fat droplets with NIH Image for all fat particles with a diameter of at least 2 µm, and found that the radii of fat droplets decreased from 3.57±0.84 µm in the vehicle control to 1.79±0.36 µm in the animals treated with 60 mg/kg/day of cerulenin (p<0.05) and 2.48±0.20 µm with 30 mg/kg/day of cerulenin **(** **Fig. 3H** **)**.

**Figure 4:**
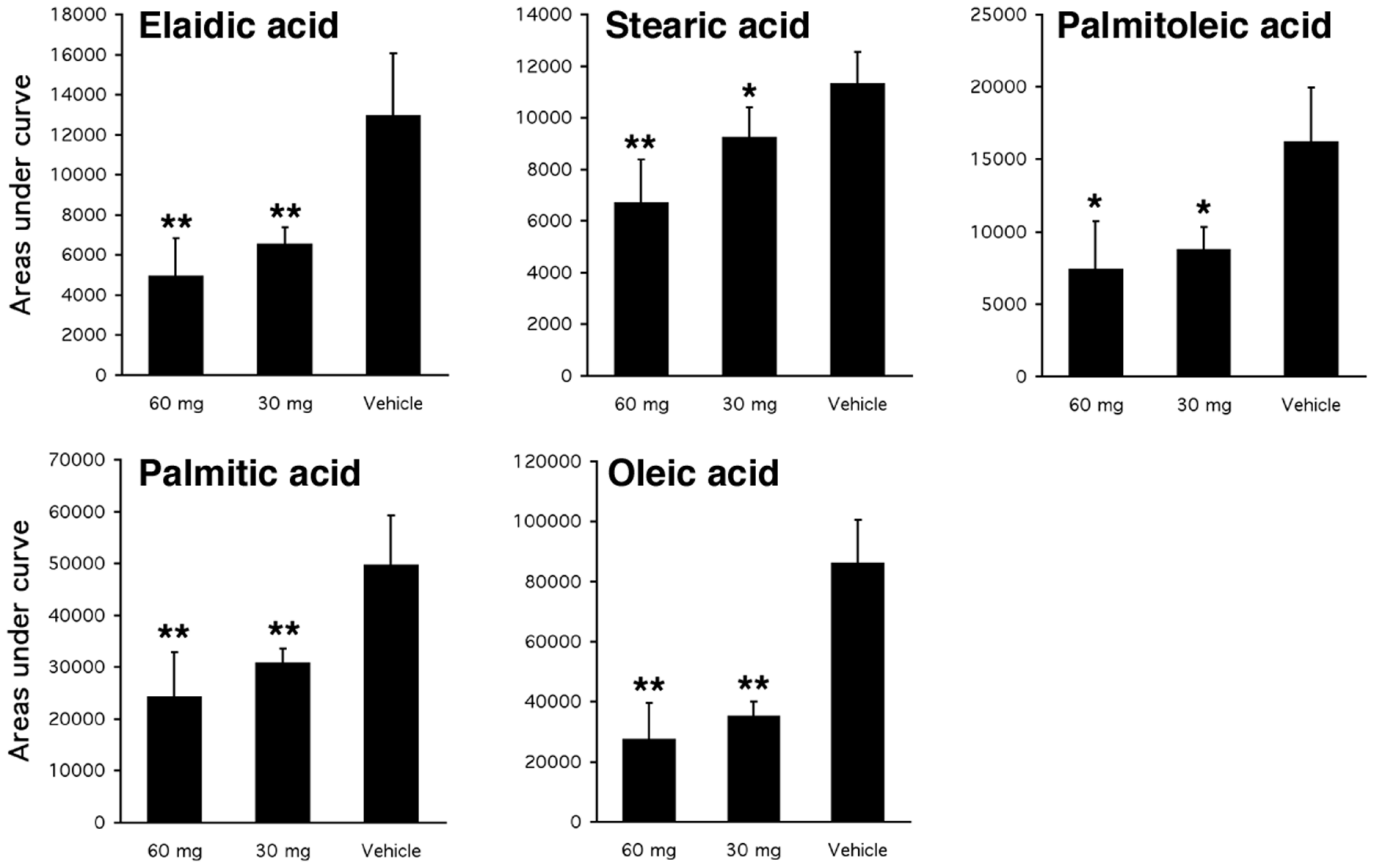
GC-FID analysis of FA content. The content of each FA is expressed as the area under curve in the GC-FID from liver samples that were treated with 7-day cerulenin.

The differences in fat content with cerulenin treatment prompted us to explore changes in the primary FA composition in these samples by gas chromatography-flame ionization detection (GC-FID). All samples with 7-day daily cerulenin treatment were analyzed and data was presented as the area under the curve of each type of FA. This analysis revealed that all FAs were not affected to the same extent by cerulenin treatment. The content of some FAs, including capric acid, myristic acid, C17 diene acid, Cis-5,8,11,14,17-eicosapentaenoic acid and arachidonic acid, were maintained at approximately the same level, while the content of others (including palmitoleic acid, palmitic acid, stearic acid, oleic/C18 acid and elaidic acid) were significantly decreased with different dosages of cerulenin treatment (**Fig. 4**).

### Cerulenin Treatment Decreases UCP2 and PPAR Expression

**Figure 5:**
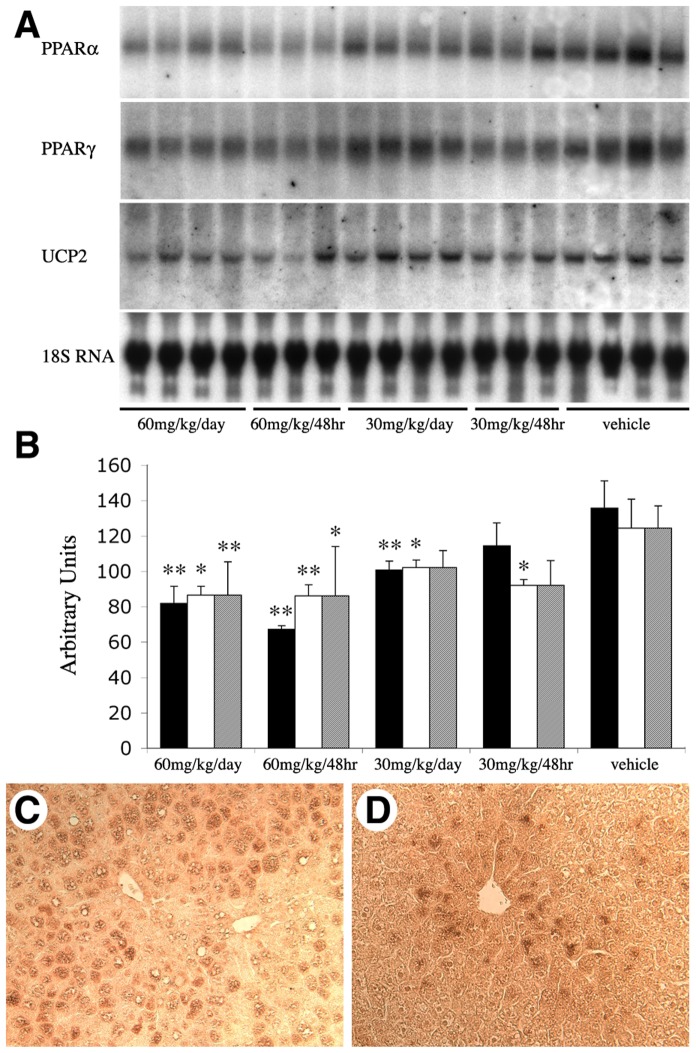
Expression of UCP2 and PPARα and γ. **A.** Northern blot analysis of PPARα, PPARγ, and UCP2**.** *ob/ob* mouse livers received 7-day (every day or every other day) treatment of cerulenin or vehicle as indicated. 18 S RNA was used for normalization. **B.** The densitometric values are expressed as averages standardized to baseline. **C (**vehicle-treated) and **D** (60 mg/kg/day for 7 days) are PPARγ immunostaining signals in *ob/ob* mouse livers, Scale bar: 60 µm.

Steatotic livers have upregulated UCP2 [9], [34], [35] and PPARα and γ [36], [37] which play a role in FA metabolism, we thus examined whether the beneficial effects of FAS inhibition on steatotic liver were mediated by the expression of UCP2 and PPARs. Consistent with the above ATP data, we found that UCP2 mRNA expression in *ob/ob* mouse livers was decreased by 7-day treatment of 60 mg/kg cerulenin, while a similar treatment using 30 mg/kg cerulenin had shown no detectable effect **(** **Fig. 5A** **)**. The same pattern of changes in the expression of PPARα and γ were also observed. Both of these signaling molecules had decreased mRNA expression with 60 mg/kg of cerulenin treatment (7 days) but not with 30 mg/kg of cerulenin **(** **Fig. 5A** **)**. Treatment with vehicle alone failed to change mRNA levels of UCP2, PPARα and PPARγ compared to unmanipulated animals (data not shown). Densitometric analysis of the mRNA levels visualized via Northern blot demonstrates that these changes were statistically significant **(** **Fig. 5B** **).** Immunohistochemical staining of PPARγ confirmed similar change at the protein level **(** **Fig. 5C&D** **)**.

### Cerulenin has a Minimal Effect on Apoptotic Activity in the Liver

**Figure 6:**
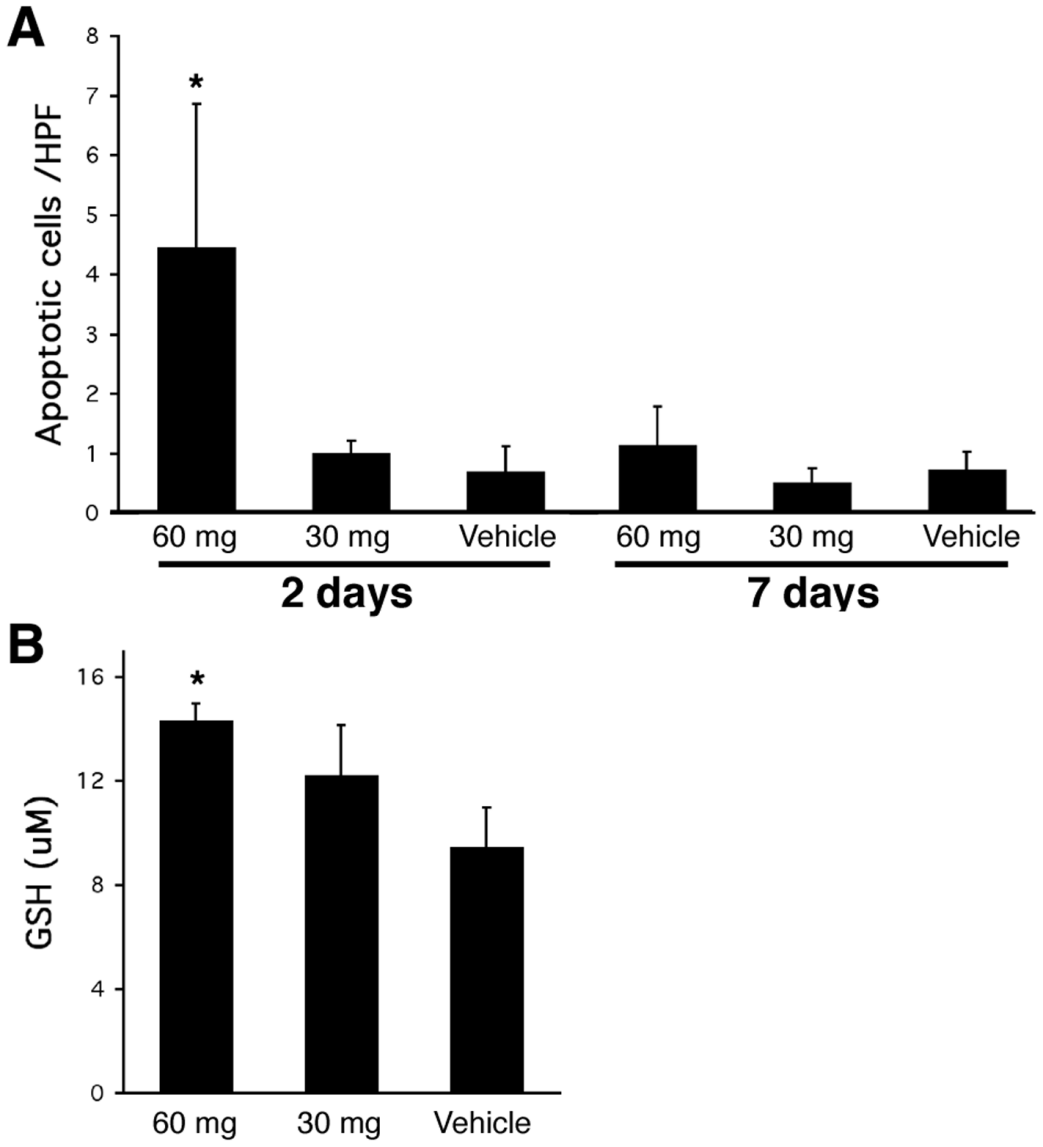
Apoptosis activity and GSH content. **A.** Apoptotic activity in *ob/ob* mouse livers with cerulenin treatment as detected by TUNEL assay. Apoptotic cells with daily cerulenin treatment as indicated in the figure were counted directly under microscope and were expressed as the number of apoptotic cells per high power field (HPF, 100 x) under light microscope. **B.** GSH content in *ob/ob* mouse livers with 7-day daily cerulenin treatment. *: *P<0.05.*

It has been demonstrated that administration of cerulenin and other FAS inhibitors in cancer tissues is cytotoxic and induces apoptosis [38], [39]. We carefully examined H&E staining of liver samples from each group, and failed to find any evidence of cytotoxicity associated with cerulenin administration. In order to clarify this question, TUNEL assay was performed in order to determine the distribution of apoptotic cells within the liver samples. We found apoptotic cells in some of the groups that received 60 mg/kg cerulenin but not in groups that received 30 mg/kg cerulenin (**Fig. 6A**). However, the number of apoptotic cells observed in samples from 60 mg/kg cerulenin treatment was very low, never exceeding a value greater than 0.1% of the total cell population. These data suggested that at the doses investigated cerulenin was not appreciably toxic to steatotic livers.

### Cerulenin Treatment does not Increase ROS Production

UCP2 reduces production of ROS within cells as a result of decreased mitochondrial membrane potential [40]. Since steatotic livers have intrinsically higher levels of ROS [7], the downregulation of UCP2 in steatotic livers by cerulenin raises a concern that this might result in an increased production of ROS. We thus measured cellular glutathione (GSH) levels in the liver from each experimental group. GSH is the major intracellular antioxidant that acts as the immediate donor of electrons to neutralize H_2_O_2_ and as a scavenger of oxygen and nitrogen-based free radicals [41]. An increased production of ROS in steatotic livers will therefore result in reduced GSH content. Vehicle alone did not change GSH content in the steatotic liver (data not shown). However, in animals treated with the high dose for 7 days (groups A and B), the GSH levels were increased 40–50% (p<0.05) (**Fig. 6B**). Values observed in the animals treated with the lower dose were also increased but the elevation was not statistically significant. Short-term treatment (2 days) of cerulenin resulted in only slightly increased GSH levels over the vehicle and baseline controls.

## Discussion

In this study, we addressed the hypothesis that excess fat plays a central role in the pathogenesis of hepatic steatosis resulting from the overexpression of PPARα, PPARγ, and UCP2. In order to address this hypothesis, cerulenin was used to determine if FAS blockade could effectively ameliorate the pathogenesis of hepatic steatosis. Blockade of FAS was found to effectively deplete fat content, increase intracellular ATP levels, and improved hepatic function. This study also suggests that these effects were mediated by suppressed expression of PPARα, PPARγ, and UCP2. We have previously demonstrated that *ob/ob* livers have decreased ATP content and increased UCP2 expression [12]. Based on the facts that UCP2 expression is increased in steatotic livers and that steatotic livers have reduced ATP content and impaired liver function, we hypothesized that overexpression of UCP2 would be harmful to steatotic hepatocytes [12], [28]. Indeed, in this study, new evidence was generated that the hepatic ATP levels are inversely related to UCP2 expression. These results, together with our previous findings, strongly suggest that overexpression of UCP2 in the steatotic liver is detrimental to normal liver function, and the protective effects of cerulenin to the fatty liver are at least partially through decreased UCP2 expression.

PPARs are nuclear receptors that bind to specific PPAR response elements in the promoter region of their target genes [42] and regulate genes involved in lipid utilization, storage and lipoprotein metabolism [43], [44], [45]. PPARγ is highly expressed in adipose tissues [46] and plays a critical role in the differentiation of adipocytes [47], [48] and in adipogenesis [49]. PPARα is a key controller of intermediary metabolism and transcriptionally regulates a number of genes involved in FA oxidation [43], [50]. It is well documented that PPARα and γ expression are upregulated by FAs [51] and that the effects of FAs on UCP2 expression regulation are through PPAR mediated pathways [52]. FAs and other ligands specific to PPARα promote UCP2 expression in neonatal cardiomyocytes [15]. Adipose tissues treated with BRL49653 and bromopalmitate, two potent activators of PPARγ, have increased UCP2 mRNA levels [53]. Mice treated with pioglitazone, a specific stimulant of PPARγ, show increased UCP2 mRNA levels in the skeletal muscle [54]. Since UCP2 upregulation inhibits ATP synthesis, we hypothesized that depleted ATP levels and impaired hepatic function in steatotic livers are triggered by excess fat, which in turn activates PPARα and γ, and upregulates UCP2. In the present study, we noticed the corresponding changes in the expressions of PPARs and UCP2, ATP and the fat content, in *ob/ob* livers treated with cerulenin, which support our hypothesis. In fact, treatment of obese and diabetic mice with the selective PPARγ activator thiazolidinediones has been reported to result in centrilobular steatosis in the liver [55], and disruption of PPARγ in *ob/ob* mice has been shown to be beneficial to the liver [56]. In our experiments, we found that with fat depletion, the expression of PPARα, PPARγ, and UCP2 were decreased, accompanied by increased ATP content and improved hepatic function. In this cascade of signaling, we believe that PPARα and γ are downstream signaling molecules of excess fat but upstream regulators of UCP2 expression. This explains why fat depletion leads to elevated ATP levels and improved liver function.

Two factors may limit the usefulness of cerulenin in improving hepatic function: increased ROS production and apoptosis. The production of ROS is positively related to mitochondrial membrane potential and the level of coupling of mitochondrial respiration to ATP production. It has been reported that UCP2 expression decreases production of ROS due to its effect on mitochondrial membrane potential [57]. Also, cerulenin treatment may lead to apoptosis in cancer tissues [58], [59]. However, controversy exists in regard to the role of UCP2 in the production of ROS. In the liver of *ob/ob* mice and in primary culture, hepatocytes produce increased amounts of ROS while the UCP2 level is high [11]. In our experiments, we did not observe any significant decrease in GSH level with cerulenin treatment. Instead, we found GSH contents significantly increased (indicating decreased production of ROS) in some cerulenin treated *ob/ob* mice when UCP2 expression was downregulated. We noted that mice with higher GSH contents had the most dramatic fat decrease. Given that excess lipid oxidation and/or excess lipid storage leads to increased ROS production [35], our results suggest that the effect of reduced UCP2 expression on ROS production is well offset by profound decreases in fat content, which help to lower ROS levels. Our data also demonstrate that cerulenin at 60 mg/kg had no obvious cytotoxic effect in the steatotic livers, and apoptotic activity was only marginally affected.

In summary, the FAS blocker cerulenin effectively depleted fat content in the steatotic liver, which triggered a series of reactions: expression of PPARα and γ were decreased resulting in lower UCP2 level and increased hepatic ATP content, and decreased ROS production. Increased intracellular ATP levels and decreased ROS ultimately improved liver function. Currently, there is no effective treatment for hepatic steatosis. We believe these findings have significant clinical importance as a potential new therapeutic strategy for hepatic steatosis in general and for steatotic livers subjected to I/R injury.
